# Comparison of Drug Release and Pharmacokinetics after Transarterial Chemoembolization Using Diverse Lipiodol Emulsions and Drug-Eluting Beads

**DOI:** 10.1371/journal.pone.0115898

**Published:** 2014-12-31

**Authors:** Jin Woo Choi, Hyun-Jong Cho, Ju-Hwan Park, Song Yi Baek, Jin Wook Chung, Dae-Duk Kim, Hyo-Cheol Kim

**Affiliations:** 1 Department of Radiology, Seoul National University Hospital, Seoul, Korea; 2 College of Pharmacy, Kangwon National University, Chuncheon, Korea; 3 College of Pharmacy and Research Institute of Pharmaceutical Sciences, Seoul National University, Seoul, Korea; Kaohsiung Medical University Hospital, Kaohsiung Medical University, Taiwan

## Abstract

In many studies for chemoembolization of hepatocellular carcinoma, the Lipiodol emulsion preparation protocols, especially the mixing steps, were unclear or even unrevealed at all. However, doxorubicin (DOX) release may depend on the composition and volume ratio (Lipiodol to DOX solution) of a Lipiodol emulsion. Therefore, we conducted a preclinical study to compare *in-vitro* drug release and *in-vivo* pharmacokinetics of DOX from diverse Lipiodol emulsions and drug-eluting beads (DEBs) and to compare the tumor response in a rabbit VX2 carcinoma model. DOX release profiles of four types of Lipiodol emulsions with different media (normal saline or Pamiray as an iodinated contrast medium), volume ratio (Lipiodol to DOX solution), and DEBs were investigated *in-vitro*. For the *in-vivo* study, 15 rabbits bearing VX2 carcinoma in the liver were treated with 4∶1 volume ratio Lipiodol emulsion (group A), 1∶1 volume ratio Lipiodol emulsion (group B), and DEBs (group C) chemoembolization. Blood and tissue sampling was conducted to evaluate DOX concentration in plasma and tissues, histological changes, and liver toxicity. The most stable emulsion was formed with Pamiray (including DOX) at a 4∶1 volume ratio. The AUC value of group A was significantly lower than that of group B (*p* = 0.003) but comparable to that of group C (*p* = 0.071). The C_max_ value of group A was significantly different compared with those of group B (*p* = 0.004) and C (*p* = 0.015). The tissue drug concentration in group A was comparable to that in group C (*p* = 0.251). No viable tumor was detected in rabbits of group A and B. In group C, viable tumor less than 10% was seen in two of the five rabbits. There were no significant differences in liver enzyme levels after the procedure. In conclusion, DOX release and pharmacokinetics of presented emulsion systems depend substantially on their composition. Therefore, Lipiodol emulsion type should be considered when interpreting data and designing new studies dealing with chemoembolization.

## Introduction

Transarterial chemoembolization (TACE) has been used globally as a palliative treatment option in patients with unresectable hepatocellular carcinoma (HCC) [1], [2]. Lipiodol (Laboratoire Andre Guerbet, Aulnay-sous-Bois, France) has been used as a drug delivery system for over 30 years and is still widely adopted for daily practice in many Asian countries because of its established clinical utility [3], [4]. Meanwhile, drug-eluting beads (DEBs) have become popular as drug carriers in Western countries in the past decade [5].

*In-vitro* studies have claimed that drug elution from DEBs is controlled and sustained, unlike the rapid drug release from Lipiodol emulsions [6], [7]. In addition, a recent clinical trial comparing the use of doxorubicin (DOX)-loaded beads versus conventional transarterial chemoembolization (cTACE) demonstrated reduced side effects when using the former despite the DEB's uncertain survival gain [8].

However, interestingly, the systemic side effects of cTACE have not been described frequently in previous literature originating from Asian countries [9], and the Lipiodol emulsion preparation protocols followed in Asian and Western countries are different [10], [11]. In addition, the Lipiodol emulsion regimen in an *in-vitro* drug release test was quite inconsistent with its common clinical usage [6]. DOX is dissolved in iodinated contrast media and mixed with Lipiodol in most institutions [12]–[15], but in the previous studies, DOX was dissolved in saline, which could have contributed to rapid separation of DOX from Lipiodol [6]. Therefore, a comparative study of drug release from DEBs and various kinds of Lipiodol emulsions is warranted.

We hypothesized that DOX release depends on the composition and volume ratio (between Lipiodol and DOX solution) of a Lipiodol emulsion, which may result in different tumor responses and side effects in patients. Therefore, we conducted a preclinical study to compare *in-vitro* drug release and *in-vivo* pharmacokinetics of DOX from diverse Lipiodol emulsions and DEBs, and to compare the tumor response in a rabbit VX2 carcinoma model. The model is commonly used in TACE researches by providing similarities with localized hepatoma of human.

## Materials and Methods

### Formulation preparation

We prepared four types of Lipiodol (Laboratoire Andre Guerbet, Aulnay-sous-Bois, France) emulsions: A) 10 mg of DOX in 0.5 ml of contrast media (Iopamidol, Pamiray 250; DongKook Pharmaceutical, Seoul, Korea) mixed with 2 ml of Lipiodol, B) 10 mg of DOX in 1.25 ml of Pamiray mixed with 1.25 ml of Lipiodol, C) 10 mg of DOX in 0.5 ml of normal saline (NS) mixed with 2 ml of Lipiodol, and D) 10 mg of DOX in 1.25 ml of NS mixed with 1.25 ml of Lipiodol. DOX was dissolved in Pamiray (A and B) and NS (C and D). The volume ratios of Lipiodol and DOX solution were 4∶1 (A and C) and 1∶1 (B and D). Emulsions composed of DOX solution (aqueous phase) and Lipiodol (oil phase) were prepared by repetitive pumping through a three-way stopcock. Photographs of these four types of Lipiodol emulsions were obtained under a light microscope (DM2500, Leica Microsystems, Wetzlar, Germany) at 1 and 10 minutes after pumping.

DEBs (DC bead; Biocompatibles, Farnham, United Kingdom) measuring 100–300 µm (for in-vitro and in-vivo studies) and 300–500 µm (for in-vitro study) in diameter were loaded with DOX according to the manufacturer's instructions (37.5 mg/ml).

### *In-vitro* release test

*In-vitro* drug release from the Lipiodol emulsions (4 types) or DEBs (2 types) was evaluated. Aliquots of DOX-loaded formulations (corresponding to 0.2 mg of DOX) were loaded into mini GeBAflex tubes (molecular weight cut-off: 12–14 kDa; Gene Bio-Application Ltd., Kfar Hanagide, Israel). Those tubes were immersed in 10 ml of phosphate-buffered saline (PBS, pH 7.4) and incubated in a shaking bath (37°C) rotated at a speed of 50 rpm. At predetermined times (0.5, 1, 2, 3, 4, 6, 8 h, and 24 h), the aliquots (0.2 ml) were collected and replaced with equivalent volumes of fresh release media.

The released amounts of DOX were determined using high performance liquid chromatography (HPLC), as reported [16]. The drug was analyzed using a Waters HPLC system (Waters Co., Milford, MA, USA) equipped with a separation module (Waters e2695), fluorescence detector (Waters 2475), and column (reverse-phase, C18, 250×4.6 mm, 5 µm; Xbridge, Waters Co.). The fluorescence of DOX was detected at wavelengths of 480 nm (excitation) and 560 nm (emission). The mobile phase was composed of 10 mM potassium phosphate buffer (pH 2.5) and acetonitrile (including 0.1% triethylamine) mixture (73∶27, v/v). The injection volume was 20 µl, and the flow rate was 1 ml/min. The lower limit of quantification (LLOQ) of the drug was 25 ng/ml. Precision and accuracy were within the acceptable range.

### In-vivo test

#### Animal experiments

This experiment was approved by the Institutional Animal Care and Use Committee of Seoul National University Hospital, and performed in accordance with the institutional guidelines. Twelve male New Zealand White rabbits weighing 3.0±0.1 kg were used for this experiment. During the whole experiments, they were raised in individual conventional cages of our experimental animal facility, and monitored twice a day by a dedicated animal experimenter (B.S.Y). The VX2 carcinoma strain was maintained by successive transplantations into the hind limb of a carrier rabbit. After the anesthesia, achieved by injecting a solution of zolazepam (5 mg/kg, Zoletil; Virbac, Carros cedex, France) and xylazine (10 mg/kg, Rompun; Bayer-Schering Pharma, Berlin, Germany) into the hindlimb, and midline abdominal incision, a tumor chip of 1 mm^3^ was directly implanted into the left medial lobe of the liver. After the tumor implantation surgery, meloxicam of 1 mg was injected subcutaneously to reduce pain, and blood oxygen saturation was monitored by using a pulse oximetry until the rabbits were fully recovered from the anesthesia. The tumors were allowed to grow for 3 weeks to form a solitary, well-demarcated tumor measuring 15–25 mm in diameter.

Three weeks after the implantation of VX2 carcinomas in the liver, an enhanced computed tomography (CT) scan was performed for measuring the tumor size. Rabbits with tumor formation failure (n = 1), tumor dissemination (n = 1), mean tumor diameter less than 15 mm (n = 2), and unintended death during anesthesia (n = 1) were excluded from the study. Rabbits with similar-sized tumors were distributed evenly in each of the following groups (5 rabbits per group) by an experimenter (B.S.Y) blinded to the TACE procedures: A) emulsion A (0.6 ml containing 2.4 mg DOX), B) emulsion B (0.6 ml containing 2.4 mg DOX), and C) DEBs 100–300 µm (1.2 ml containing 2.4 mg DOX). Adjuvant embolization was conducted for groups A and B by using polyvinyl alcohol particles (Contour; Boston Scientific, Natick, MA) of size 150–250 µm to simulate the popular cTACE method in clinical practice and to mimic the embolic effect of DEBs (100–300 µm).

TACE was performed under fluoroscopic guidance by an experienced interventional radiologist (K.H.C.). After a 4-Fr sheath was inserted through the femoral artery, celiac angiography was performed for identifying tumor staining by using a 1.7-Fr microcatheter (Excelsior SL-10; Boston Scientific, Natick, MA). After the microcatheter was advanced to the left hepatic artery, the chemotherapeutic agent was injected slowly. Blood samples were obtained at 0, 2, 5, 10, 20, 30, and 60 min after injection in all animal groups for determining pharmacokinetics. Plasma was collected by centrifugation and stored at −70°C prior to quantitative drug analysis.

To determine the hepatic toxicities caused by TACE, blood sampling was performed at 0, 1, 3, and 7 days after the procedure. Plasma aspartate aminotransferase (AST) and alanine aminotransferase (ALT) levels in the samples were measured using a biochemical autoanalyzer. Afterwards, the presence of adverse effects of DOX such as hair loss and diarrhea was checked daily.

Seven days after the procedure, the rabbits were evaluated by enhanced CT scans and then euthanized in a carbon dioxide chamber for further analyses. Thereafter, their livers were explanted and sectioned in 5 mm slices.

#### DOX concentration in plasma

DOX concentration in plasma was analyzed using HPLC assay. Plasma samples (150 µl) were mixed with 10 µg/ml propranolol solution, as an internal standard (IS), in distilled water (25 µl). Acetonitrile (550 µl) was added to that mixture, and the resulting mixture was vortexed and centrifuged for 5 min at 13,200 rpm. After centrifugation, the supernatant was collected, and the organic solvent was evaporated under a nitrogen gas stream at 50°C. This tube was resuspended with a mobile phase (60 µl), and 20 µl of the supernatant obtained by centrifugation was injected for quantitative analysis of the drug. The analytical condition of DOX was the same as that in the described HPLC method. The wavelengths of fluorescence detection of propranolol (IS) were 230 nm (excitation) and 340 nm (emission). Moreover, the precision and accuracy values were within the acceptable ranges.

Drug concentration in plasma at each time point was determined and the pharmacokinetic parameter, total area under the plasma concentration–time curve from time zero to infinity (AUC) was calculated using WinNonlin software (ver. 3.1, Pharsight; Mountain View, CA, USA). The peak drug concentration in plasma (C_max_) was directly read from the experimental data.

#### DOX concentration in liver

DOX concentrations in the normal liver and tumor regions were determined by the described HPLC method. The liver and tumor were dissected separately. Especially, the tumor was dissected into two equal halves. One half was then used for DOX analysis according to the described HPLC method, and the other half was submitted for histological evaluation. For measuring DOX concentrations in the liver and tumor regions, each tissue was mixed with PBS (1∶5, v/v) and homogenized. The subsequent pretreatment method was the same as that for plasma.

#### Histological analyses

The extent of infarction of the normal liver was visually assessed and was graded as mild (less than 10% infarcted volume in the normal liver), moderate (10–30%), and severe (more than 30%) infarction.

Hematoxylin and eosin (H&E) staining, and terminal deoxynucleotidyl transferase dUTP nick end labeling (TUNEL) staining were performed on the paraffin-embedded sections.

### Statistical analysis

The Kruskal-Wallis test was used for determining statistical significance between the groups. *P*<0.05 was considered statistically significant. Software Package for Statistical Analysis (SPSS 21.0; SPSS, Chicago, IL) was used for this purpose.

## Results

### *In-vitro* drug release test

**Figure 1:**
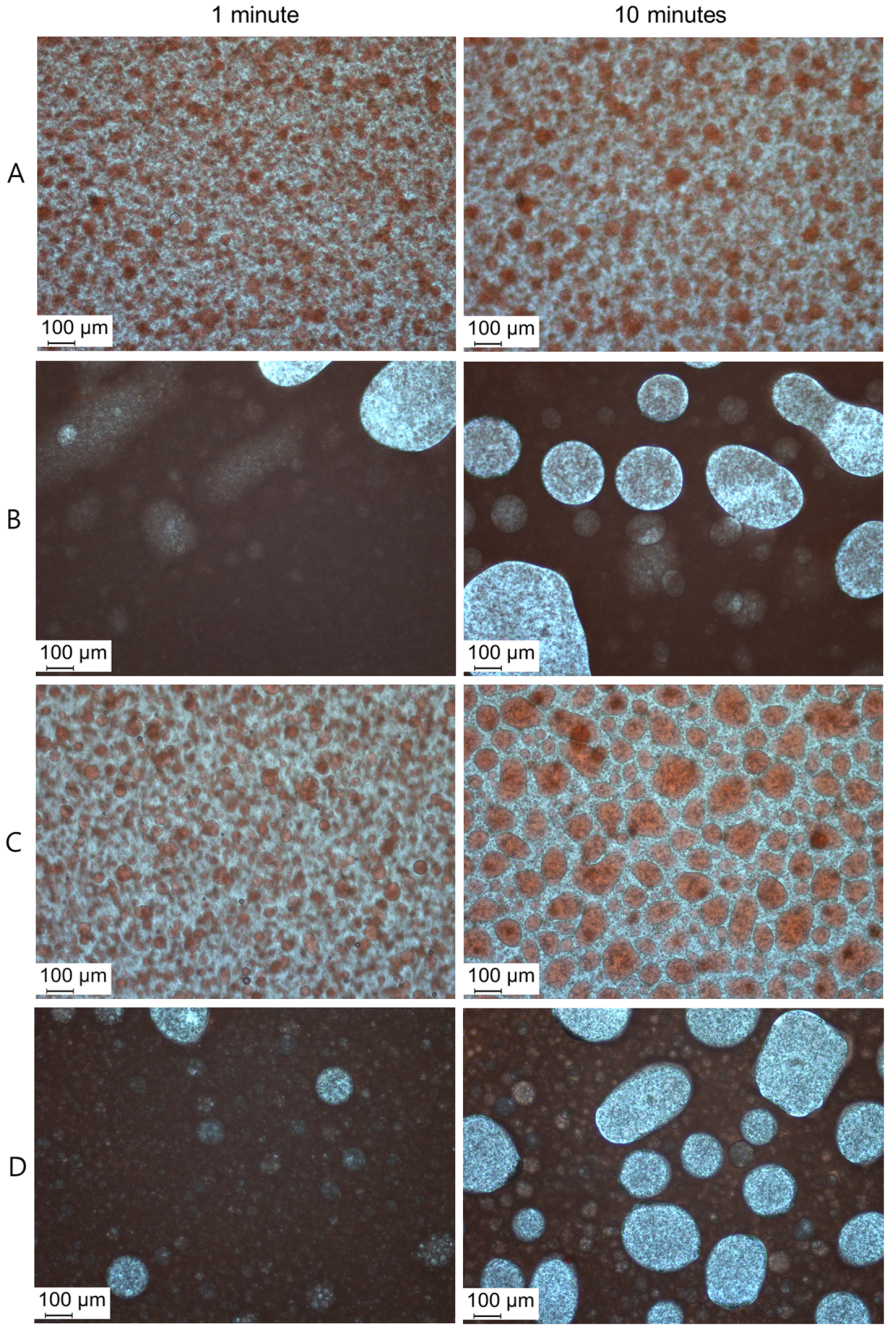
Photograph of four types of Lipiodol emulsions: (A) 10 mg of DOX in 0.5 ml of Pamiray mixed with 2 ml of Lipiodol, (B) 10 mg of DOX in 1.25 ml of Pamiray mixed with 1.25 ml of Lipiodol, (C) 10 mg of DOX in 0.5 ml of NS mixed with 2 ml of Lipiodol, (D) 10 mg of DOX in 1.25 ml of NS mixed with 1.25 ml of Lipiodol. Left images were obtained 1 minute after pumping and right images were obtained 10 minutes after pumping. Most stable w/o emulsion was prepared by 4∶1 volume ratio of Lipiodol and DOX solution in Pamiray (A).

Stable water-in-oil (w/o) emulsions were created using a 4∶1 volume ratio of Lipiodol and DOX solution in Pamiray (A) or NS (C). Particularly, emulsion A formed the most stable and even droplets (Fig. 1). In contrast, the emulsions created using a 1∶1 volume ratio of Lipiodol and DOX solution in Pamiray (B) or NS (D) demonstrated rapid separation of water and oil, within 10 min after mixing (Fig. 1).

**Figure 2:**
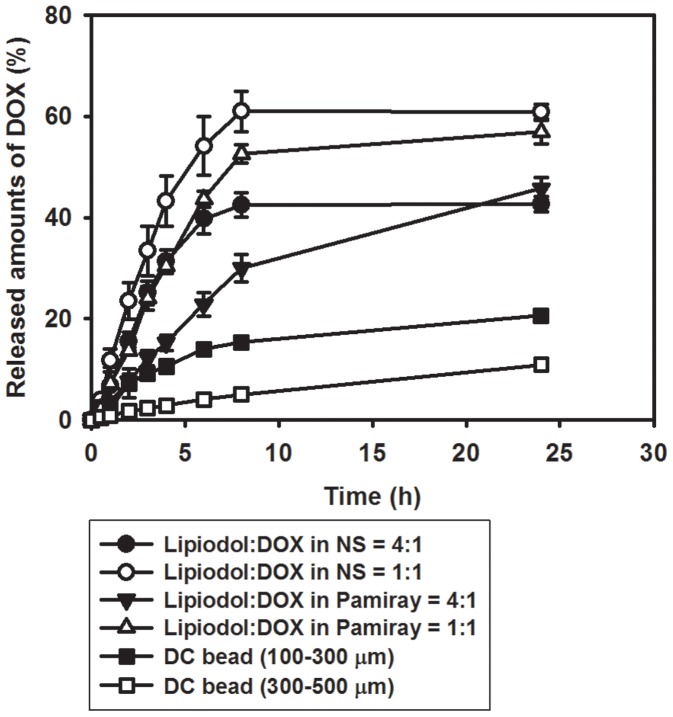
DOX release profiles from four types of emulsion systems and DEBs. Each point indicates mean ± standard deviation (SD) (n = 4).

The DOX release profiles of the four different Lipiodol emulsions and DEBs are shown in Fig. 2. The released amounts (%) of DOX at 8 h are as follows: 5.08±0.27% for DEBs 300–500 µm, 15.34±0.33% for DEBs 100–300 µm, 29.96±2.70% for emulsion A, 52.58±1.81% for emulsion B, 42.53±2.40% for emulsion C, and 61.00±3.97% for emulsion D. Lipiodol-based emulsions exhibit rapid drug release compared with DEBs. Sustained drug release profiles were observed for the 4∶1 volume ratio (between Lipiodol and DOX solution) rather than the 1∶1 volume ratio. Regardless of the volume ratio (1∶1 or 4∶1), the Pamiray-based emulsions exhibited sustained drug release profiles compared with the NS-based emulsions (Fig. 2). Therefore, Pamiray-based emulsions containing DOX and Lipiodol were selected for further *in-vivo* studies.

### In-vivo test

#### DOX concentration in plasma

**Figure 3:**
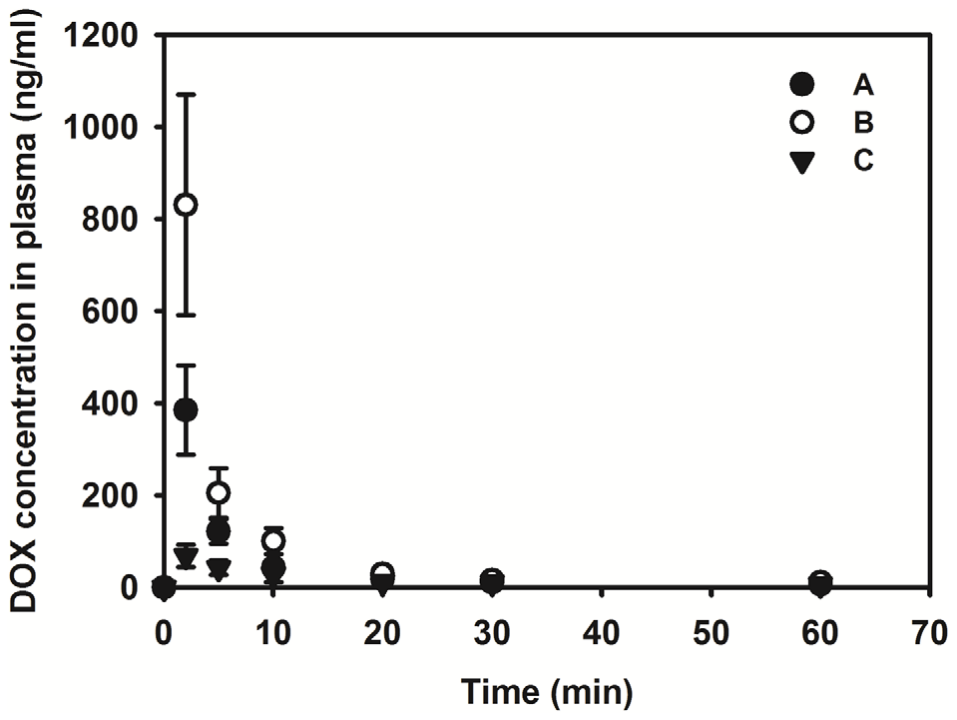
DOX concentration in plasma after intraarterial administration of emulsions composed of Lipiodol and DOX in Pamiray (A and B) and DEBs 100–300 µm (C). Each point indicates mean ± SD (n≥3).

There rabbits bearing VX2 carcinomas in the liver weighed 3.7–4.1 kg. There were no significant differences among the mean weight of the three groups (5 rabbits per group). DOX concentrations in plasma after intraarterial administration of emulsions with Pamiray as the aqueous phase (group A with 4∶1 volume ratio and group B with 1∶1 volume ratio), as well as DEBs (group C), in the rabbits are shown in Fig. 3, and the AUC and C_max_ values are summarized in Table 1. The AUC value, indicating systemic exposure to a drug, of group A was significantly lower than that of group B (*p* = 0.003), but there was no significant difference with that of group C (*p* = 0.071). The AUC value of group B was 3.43-fold higher than that of group C (*p*<0.001). The C_max_ value of group A exhibited a significant difference compared with the values of group B (*p* = 0.004) and group C (*p* = 0.015). Particularly, the C_max_ value of group B was 12.12-fold higher than that of group C (*p*<0.001).

**Table 1:** Pharmacokinetic parameters of DOX after intraarterial injection in rabbits at a dose of 0.8 mg/kg.

Parameters	Group A (Lipiodol:DOX in Pamiray = 4∶1)	Group B (Lipiodol:DOX in Pamiray = 1∶1)	Group C (DEBs 100–300 µm)
AUC (ng•min/ml)	2409.58±326.42*	4612.51±1044.78+	1346.51±398.39
C_max_ (ng/ml)	384.99±96.44* ^,^ +	830.30±239.37+	68.48±23.87

*p<0.05 compared to Lipiodol:DOX in Pamiray  = 1∶1 group (group B).

+ *p*<0.05 compared to DEBs group (group C).

T_max_ value of all groups was 2 min.

Data present as mean ± SD (*n*≥3).

Post hoc: scheffe.

#### DOX concentration in liver

DOX concentrations in the normal liver and tumor regions 7 days after TACE are listed in Table 2. In all groups, DOX concentration in the tumor region was much higher than that in the normal liver region. The mean values of DOX in the normal liver and tumor region at 7 days post injection were as follows: group B< group A< group C. Nonetheless, in this study, the drug concentration in the tumor region on day 7 in group A was comparable to that in group C (*p* = 0.251). The tumor-region DOX concentration of group B was significantly lower than that of group C (*p* = 0.021).

**Table 2:** Drug concentration (ng/g in tissues) in normal liver and tumor region after intraarterial injection in rabbits at a dose of 0.8 mg/kg.

Region	Group A (Lipiodol:DOX in Pamiray = 4∶1)	Group B (Lipiodol:DOX in Pamiray = 1∶1)	Group C (DEBs 100–300 µm)
Tumor	6749.7±2472.6	1736.5±331.3+	10240.0±5271.1
Normal liver	149.9±85.8	25.1±7.8	198.1±190.0

+ *p*<0.05 compared to DEBs group (group C).

Rabbits were sacrificed 7 days postinjection and drug concentrations in the tissues were determined.

Data present as mean ± SD (*n*≥3).

Post hoc: LSD.

#### Histological analyses

**Figure 4:**
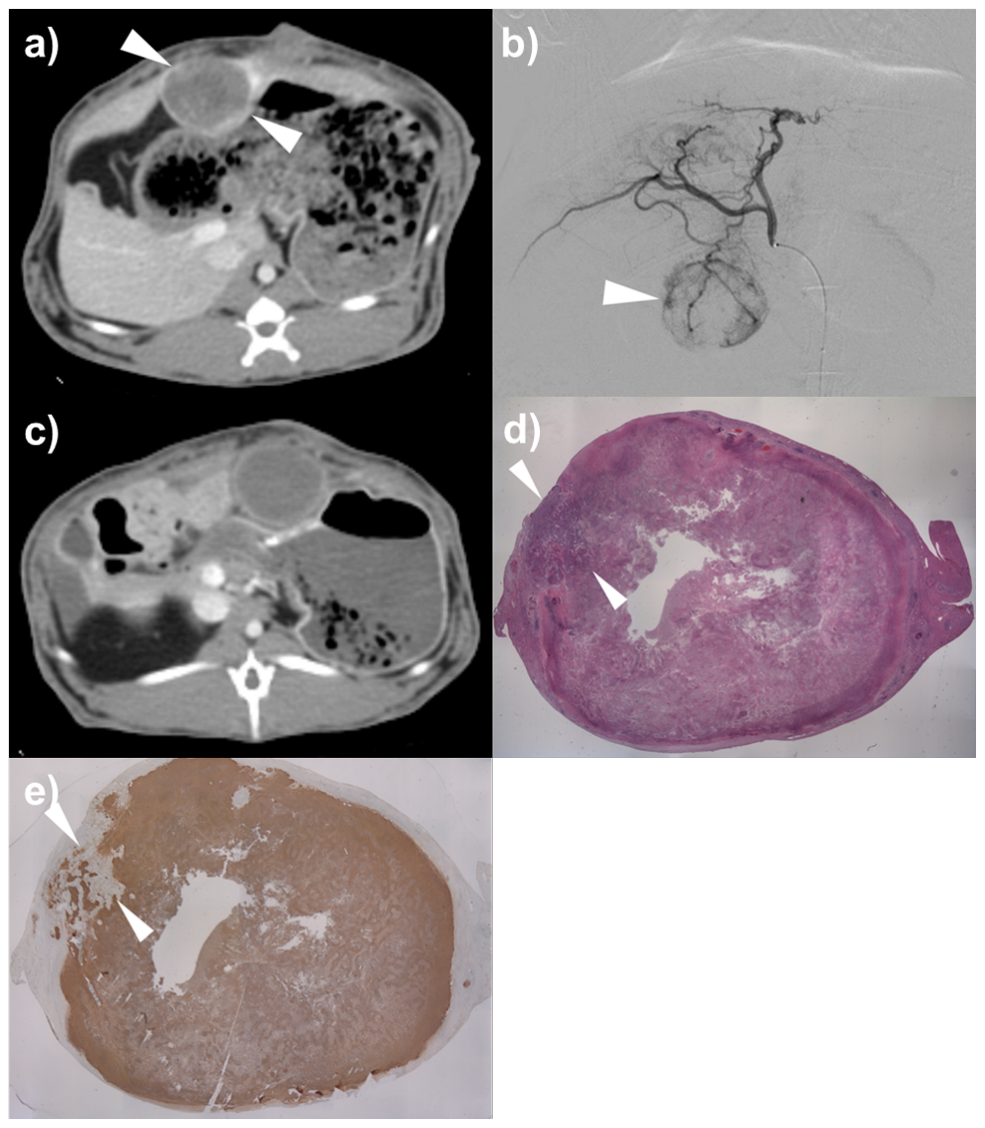
The procedural steps of *in vivo* experiment using VX2 rabbit liver tumor model. a) CT scan of portal venous phase showed a well-demarcated solitary tumor in the left hepatic lobe of the rabbit (arrowheads). b) Common hepatic angiography shows a hypervascular tumor staining (arrowhead). c) CT scan of portal venous phase 1 week after TACE using drug-eluting beads demonstrates no enhancement within the tumor (arrowheads). d) Photomicroscopic slide of the tumor specimen indicates focal viable tumor (arrowheads) (Hematoxylin-Eosin staining, X1). e) TUNEL staining indicates focal viable tumor (arrowheads).

Tumor size (mean ± standard deviation) of groups A, B, and C were 2.18±0.29 cm, 2.16±0.52 cm, and 2.1±0.41 cm, respectively (*p* = 0.906). On gross examination, focal liver infarction was evident in three rabbits of group A, five rabbits of group B, and one rabbit of group C (Table 3). On histological examination, no viable tumor was detected in all rabbits of group A and B. In group C, viable tumor less than 10% was seen in two rabbits, and no viable tumor was detected in three rabbits (Fig. 4).

**Table 3:** Results of histologic examinations.

		Group A (Lipiodol:DOX in Pamiray = 4∶1)	Group B (Lipiodol:DOX in Pamiray = 1∶1)	Group C (DEBs 100–300 µm)
Parenchymal necrosis	No	2	0	4
	Mild	1	1	1
	Moderate	2	4	0
Viable tumor	Absent	5	5	3
	Present	0	0	2

#### Liver toxicity and adverse effect

**Figure 5:**
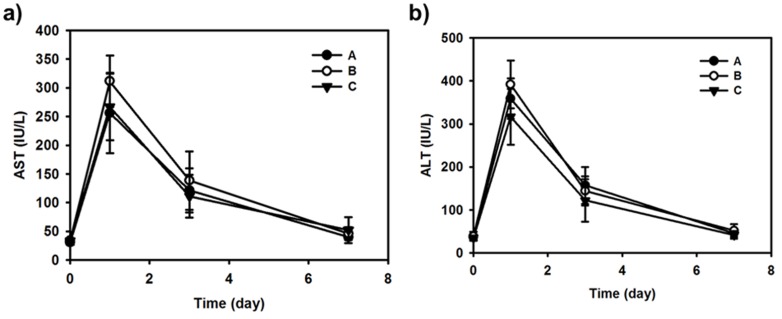
Hepatotoxicity measurement after intraarterial administration of formulations (A, B, and C). Each point indicates mean ± SD (n = 5). a) AST value profiles according to the time. b) ALT value profiles according to the time.

In all groups, the plasma AST and ALT levels were elevated 1 day after TACE, but decreased to the near-normal range at 7 days after the procedure (Fig. 5). Differences among the groups were not significant (*p*>0.100, Kruskal-Wallis test). During the follow-up period, adverse effects of DOX such as hair loss or diarrhea were not observed in all rabbits.

## Discussion

We demonstrated that both aqueous phase type (NS or Pamiray) and volume ratio (1∶1 or 4∶1) between oil and aqueous phase can determine the stability of an emulsion system and its *in-vivo* performance. As shown in Fig. 1, the emulsion with 4∶1 volume ratio between the oil and aqueous phases exhibited better droplet size homogeneity compared with the emulsions having 1∶1 volume ratio. In emulsions with 1∶1 volume ratio (Fig. 1B and D), larger droplets were observed, probably owing to higher surface tension or coalescence. Moreover, DOX dissolved in iodinated contrast media (Pamiray in this study) formed more stable emulsions with Lipiodol than DOX dissolved in NS. According to Fig. 1A and C, the droplet size of the emulsion system composed of Pamiray containing DOX and Lipiodol was unchanged after 10 min. An emulsion system based on Pamiray (rather than NS) as the water phase and 4∶1 volume ratio between Lipiodol and the contrast media (rather than 1∶1) provided better physical stability and sustained drug release (Fig. 2). These properties seemed to contribute to the lower systemic exposure and enhanced tumor uptake of the drug after intraarterial administration in a liver tumor model (Fig. 3 and Tables 1 and 2). Unlike group B, group A exhibited no statistical difference in the AUC value and drug concentration in the liver tumor region compared to group C. These improved pharmacokinetic properties of group A may also be related to similar unwanted systemic toxicity and comparable anticancer effect in the DEB-TACE treatment group (group C) (Figs. 4 and 5).

TACE is playing a greater role in clinical practice owing to the increasing incidence of HCC [1], [2] and recent advances in drug delivery system [17], [18]. However, unfortunately, cTACE protocols are not yet unified in spite of its long history in human practice, thus making it difficult to address the efficacy and safety aspects of the procedure. This problem is gaining significance in the light of the results of recent comparative studies between cTACE and DEB-TACE [8], [9], [19]–[21]. From the results of such studies, the strengths of DEB-TACE seem to be its lower systemic and hepatic toxicity, particularly related with sustained drug release [19], [20].

However, the results should be interpreted carefully, considering the following three points. Firstly, although the composition and concentration of Lipiodol emulsions plays a key role in drug release, most clinical studies that have compared cTACE and DEB-TACE have not carefully considered the type of Lipiodol emulsions used. The Lipiodol emulsion preparation protocols, especially the mixing steps, were unclear in some studies [8], [19], [21], or even unrevealed in some trials [9], [20]. In an earlier study comparing DOX infusion and cTACE, Johnson et al. [22] claimed that there was no difference with regard to the pharmacokinetic parameters of intraarterial injection of DOX/Lipiodol emulsion and intravenous administration of DOX. In this study, DOX was dissolved in NS, and the volume ratio of Lipiodol to DOX solution was 2∶5 [22]. Lewis et al. [6] also reported on the issue of the rapid separation of DOX from Lipiodol when DOX was dissolved in NS and the volume ratio of Lipiodol to DOX solution was 1∶1. Because of the unstable nature of NS-based emulsions, in many institutes, DOX has been dissolved in iodinated contrast media for sustained drug release from Lipiodol emulsion [12]–[15]. The PRECISION V study, which used 1∶1 volume ratio of DOX solution to Lipiodol (diluted up to 1∶4 only in the case of backflow) claimed that DEB-TACE was associated with lower toxicity levels than cTACE [8], [19]. However, given that the stability of Lipiodol emulsions is greatly influenced by the volume ratio of Lipiodol to DOX solution [23], [24], the toxicity profile of cTACE can be varied by modifying the volume ratio of Lipiodol emulsion. In our study, an emulsion with 4∶1 volume ratio (Lipiodol to DOX solution) showed better pharmacokinetic properties than a 1∶1 emulsion. Concordantly, experimental studies demonstrated that an excessive volume of Lipiodol over DOX solution resulted in stable w/o emulsion with increased Lipiodol uptake in tumor and higher drug delivery efficiency [25], [26]. Moreover, this can explain why hepatotoxicity and alopecia after cTACE, relatively common in some studies [13], [19], [27], have not been frequently reported in other studies using a more stable Lipiodol emulsion, *i.e.* 4∶1 (Lipiodol to DOX solution, o/w) [9]–[11].

Secondly, the methods of adjuvant embolization in cTACE are heterogeneous in previous studies [8], [19]–[21]. Adjuvant embolization may influence drug release as well as the embolic effect, considering that diminished (or eliminated) forward flow can prevent drug washout from the embolized region. In this study, no viable tumor was detected on all rabbits treated with cTACE (groups A and B), but viable tumor was observed in two rabbits treated with DEB-TACE (group C). Considering that adjuvant embolization is widely used in many institutes and that TACE efficacy is determined by the combination of cytotoxicity and the embolic effect, diverse cTACE strategies using a stable Lipiodol emulsion can be comparable to DEB-TACE.

Thirdly, many previous studies used larger-sized DEBs, ranging from 300 µm to 700 µm [8], [19]–[21], although the recent trend is to use smaller beads, measuring 100–300 µm in diameter. Given that the smaller beads demonstrate faster drug release [6], [28], the greater discrepancy of pharmacokinetic profiles between Lipiodol emulsion and larger DEBs in the previous studies [20] can be diminished by using smaller DEBs in accordance with the current trend.

Our study has a few limitations. Firstly, as a preclinical study, we used a rabbit model bearing VX2 carcinoma in the liver. Although this tumor model has been widely accepted for studying TACE, there are some discrepancies between the model and human HCC. VX2 carcinoma is not derived from hepatocytes, and the tumor is quite notorious for its propensity to necrosis. Based on our results, VX2 carcinoma may be prone to ischemic insult, which may result in sufficient therapeutic effect regardless of the TACE method. Secondly, owing to the characteristics of animal studies, subjective side-effects in daily practice, such as nausea and fatigue, could not be evaluated. Therefore, our results need to be validated further in human studies. Thirdly, only 1∶1 and 4∶1 volume ratios of Lipiodol to DOX solution are evaluated in our study. Other emulsion types such as 2∶1 or 3∶1 ratios need to be evaluated in terms of diversity in the strategies to mix Lipiodol emulsions in practice.

In conclusion, drug release from Lipiodol emulsions depends substantially on the emulsion composition and volume ratio. Therefore, Lipiodol emulsion type should be considered when interpreting data and designing new studies dealing with cTACE.
